# Transcriptomic Analysis Provides New Insights into the Tolerance Mechanisms of Green Macroalgae *Ulva prolifera* to High Temperature and Light Stress

**DOI:** 10.3390/biology13090725

**Published:** 2024-09-16

**Authors:** Kifat Jahan, Mst Shamim Ara Supty, Jun-Seok Lee, Keun-Hyung Choi

**Affiliations:** Department of Earth, Environmental and Space Sciences, Chungnam National University, 99 Daehak-ro, Yusung-gu, Daejeon 34134, Republic of Korea; kifatjahan170@gmail.com (K.J.);

**Keywords:** green algae, transcriptomics, temperature-light stress, genes, pathways

## Abstract

**Simple Summary:**

*Ulva prolifera* is an important species with a large market value. This species thrives at 10–20 °C but struggles at lower temperatures. Research has demonstrated that environmental stresses, such as temperature and light intensity, have a substantial impact on its growth through changing pathways and gene expression. Our present study examines the processes by which *U. prolifera* responds to combined high light intensity/temperature challenges, and the results point to the involvement of several genes and pathways in these intricate adaptive mechanisms. Gaining an understanding of these systems is essential to enhancing the efficiency and sustainability of macroalgae farming, particularly in light of climate change.

**Abstract:**

Our research focused on understanding the genetic mechanisms that contribute to the tolerance of *Ulva prolifera* (Chlorophyta), a marine macroalgae, to the combined stress of high temperature and high light intensity. At the mRNA level, the up-regulated DEGs showed enrichment in pathways related to ribosomes, proteasomes, and peroxisomes. The spliceosome pathway genes were found to be vital for *U. prolifera*’s ability to adapt to various challenging situations in all the comparison groups. In response to elevated temperature and light intensity stress, there was a significant increase in genes and pathways related to ribosomes, proteasomes, and peroxisomes, whereas autophagy showed an increase in response to stress after 24 h, but not after 48 h. These findings provide novel insights into how *U. prolifera* adapts to elevated temperature and light stress.

## 1. Introduction

*Ulva prolifera*, O.F. Müller, 1778, [1] (Chlorophyta) is a primary species responsible for green tide occurrence and has significant economic value as a seaweed species [2,3]. *U. prolifera* has been widely utilized by researchers as a significant ecological indicator for studying the effects of temperature and light intensity tolerance. Studies have shown that the growth of *U. prolifera* is significantly influenced by seawater temperature [4,5,6]. It thrives within a temperature range of 10–20 °C; however, its growth is inhibited when the temperature drops below 10 °C [7,8,9]. Experimental tests have revealed that various pathways and genes are differentially expressed in response to environmental stress and that these factors are closely linked to the tolerance of *U. prolifera* to temperature and light intensity stress [9,10,11,12,13,14]. Recent studies by Gu et al. (2022) and Fan et al. (2018) [2,15] examined the effects of temperature and light stress on various aspects of *U. prolifera*, including metabolic activities, biological functions, mRNA expression levels, and pathways. These studies highlight the ability of *U. prolifera* to adapt to temperature and light stress at different genetic and physiological levels [16,17]. For instance, it has been found that the expression of calcium-dependent protein kinase and Ca^2+^-related genes is affected by abiotic stress [18]. Additionally, the expression of *PCNA* and *CyclinA* was increased, and the MAPK signaling pathway was activated [19]. Furthermore, the expression of superoxide (APX) [20]. Nevertheless, the precise genetic mechanism underlying *U. prolifera* tolerance to the combined effects of high temperature and high light intensity stress remains largely unexplored. Therefore, it is essential to examine the combined effects of high temperature and light intensity stress on the molecular mechanisms of *U. prolifera* adaptation to improve our understanding of the adaptive biology of *U. prolifera*.

Having a deep understanding of how green macroalgae *U. prolifera* adapts to rapidly changing environments has important implications for improving the efficiency and sustainability of macroalgae cultivation [21]. Transcriptomic investigations are valuable in detecting instances of positive selection in many species. This information can then be utilized to employ gene editing techniques to produce the desired phenotype in the species under investigation [22]. Gaining this significant information will enhance our understanding of macroalgae cultivation and conservation [23]. Transcriptomic studies help to understand various genetic abnormalities and variations in stress-tolerance levels. These findings can be utilized to create a useful framework for enhancing the health and stress tolerance of macroalgae [24]. In addition, transcriptome sequencing enhances genomic resources to enhance tolerance to environmental stresses [25] and promotes the well-being of marine macroalgae, which is crucial for sustainable aquaculture practices [26]. The present study specifically examines a wide range of genes and pathways implicated in the response of *U. prolifera* to the combined stress of temperature and light intensity. The findings of our study can be utilized to enhance seaweed cultivation on an industrial scale and capture consumers globally. In this current era of global climate change, it is imperative to prioritize the implementation of sustainable seaweed cultivation and improve management practices [27,28,29]. Thus, further genetic study and advancement are necessary to enhance the cultivation of *U. prolifera*.

The aims of this study are to determine the degree to which various pathways and genes are expressed differently in response to changes in temperature and light intensity at different time points and to verify the hypothesis that the adaptation of *U. prolifera* is a complex process that involves the active involvement of multiple genes and pathways. This study contributes to our knowledge of the molecular-level mechanisms by which *U. prolifera* can withstand the stress caused by temperature and light intensity.

## 2. Materials and Methods

### 2.1. Algal Strain Collection and Culture Conditions

*Ulva prolifera* specimens were collected on 16 December 2022 from the subtidal zone of the coastal area of Garorim Bay, located on the west coast of South Korea (126.380798 E, 36.884541 N). After the samples were collected, they were immediately placed on dry ice and transported to the laboratory within three hours. In the laboratory, the samples were washed with ddH_2_O to remove other algae, planktonic microorganisms, and other debris. As the sample size was relatively small (100 g), different co-cultured species were manually separated with forceps and spatula. The sample was then acclimated to filtered seawater at a temperature of 18 °C, water salinity of 32, water pH of 7.9, and light intensity of 100 µmol photons m^−2^ s^−1^. The acclimation period followed a 12:12 h day:night cycle. To remove any leftover contaminants or co-culture species, we later moved little sections of the seaweed to sterilized and filtered seawater several times. Throughout the process, constant observation was carried out.

### 2.2. Temperature Treatment and Tissue Sampling

*Ulva prolifera* (3 g) was divided into three separate treatment groups: medium-high (MH), high (H), and control (C). Sterilized equipment was used during the process of splitting the algae into treatment groups. Each replicate was cultivated separately in conical flasks containing 200 mL of filtered and sterilized, and each flask was subjected to various treatment conditions. The two groups experiencing stress (MH and H) were subjected to medium-high temperature and light intensity stress (25 °C and 200 µmol photons m^−2^ s^−1^) and high-temperature and light intensity stress (30 °C and 300 µmol photons m^−2^ s^−1^), respectively. We selected the experimental temperature and light based on previously published data [25,26] and pre-experiments that we conducted in our laboratory. The control group (C) was maintained at 20 °C and light intensity of 100 µmol photons m^−2^ s^−1^. Each group had two replicates, and each conical flask contained 2 g of algae. During the experiment period, we maintained the water salinity of 32, water pH of 7.9, and 12:12 h day:night cycle. Upon completion of stress exposure, 0.1 g of algae from each group at each sampling point were collected at 24 h and 48 h, frozen in liquid nitrogen, and preserved at −80 °C for subsequent RNA extraction.

### 2.3. RNA Extraction

Tissue samples (0.1 g each) from each replication were used to extract total RNA using TRIzol reagent (Invitrogen, Carlsbad, CA, USA) following the manufacturer’s protocol. The frozen samples were pulverized using a mortar and liquid nitrogen. The quantity, purity, and integrity of RNA were assessed using a Nano Photometer spectrophotometer (Implen, Westlake Village, CA, USA) and by conducting electrophoresis on 1% agarose gels. The Qubit RNA Assay Kit was utilized to quantify the RNA concentration using a Qubit 2.0 Fluorometer (Life Technologies, Carlsbad, CA, USA). The concentration of total RNA employed for cDNA synthesis was 200 ng μL^−1^. The RNA samples were subjected to reverse transcription using a complementary DNA (cDNA) synthesis kit (Takara, Tokyo, Japan). The resulting cDNA product was kept at −20 °C until further use.

### 2.4. Transcriptome Sequencing and Quality Control

The quality of overall reads, number of total bases, number of total reads, GC content (%), and basic statistics were computed (Table S1). In order to address biases in the analysis, various artifacts, including poor-quality reads, adaptor sequences, contaminating DNA, and PCR duplicates, were removed. Trimmed data from all samples were combined into a single file to create a transcriptome reference. The Trinity program TMM (https://github.com/trinityrnaseq/trinityrnaseq/wiki: accessed on 18 September 2023) [30] was used to assemble the merged data into transcripts with de novo assembly and to remove redundancy. The longest contigs among the contigs were grouped together using the program to form non-redundant transcripts known as unigenes. The obtained unigenes were utilized for further annotation, ORF prediction, and expression analysis. To evaluate the transcriptome data and acquire a qualitative and quantitative gene expression database, we constructed 10 *U. prolifera* cDNA libraries. These libraries represented different groups subjected to high temperature and light intensity stress (H24, H48), medium-high temperature and light intensity stress (MH24, MH48), and control conditions (C0). Each group consisted of two replicates. The libraries were subjected to Illumina paired-end sequencing to identify representative transcripts associated with various biological activities. For library construction, 100 ng of RNA per sample was used as the input material for de novo transcriptome sequencing without relying on a reference genome sequence. The RNA-seq library was created using TruSeq Stranded Total RNA with Ribo-Zero Plant kit (Illumina, San Diego, CA, USA) following the manufacturer’s guidelines.

### 2.5. Assembly and Annotation

The raw sequences were submitted to the National Center for Biotechnology Information (NCBI) Short Read Archive (SRA) database and assigned accession numbers PRJNA1061775, PRJNA1061781, PRJNA1062022, PRJNA1092455, and PRJNA1062028. The database can be accessed at https://www.ncbi.nlm.nih.gov/sra: accessed on 6 January 2024 During this stage, contigs are combined to form non-repetitive distinct transcripts that are as long as feasible. These transcripts were then grouped into unigenes with a minimum length of 200 bp. Downstream analysis was conducted exclusively utilizing high-quality reads with a Q30 score or higher. In order to annotate the clustered unigenes, BLASTX (version 2.1.6) and BLASTN (version 2.13.0) analysis was performed against public databases, including Gene Ontology (GO), UniProt, NCBI non-redundant protein (NR), Pfam, EggNOG, NCBI Nucleotide (NT), and Kyoto Encyclopaedia of Genes and Genomes (KEGG). The E-value threshold used in this analysis was 10^5^. Protein-coding areas inside the unigenes were identified using open reading frame (ORF) prediction. The unigenes were subjected to read alignment, and their abundance was derived as the read count from the alignment.

### 2.6. Differential Expression Analysis

The read count value of the contigs measured by RNA-Seq by Expectation-Maximization (RSEM) (http://deweylab.github.io/RSEM/: accessed on 24 October 2023) [31] was used as the original raw data. As part of the data preprocessing stage, transcripts of poor quality were eliminated, with subsequent Trimmed Mean of M-values (TMM) (http://www.usadellab.org/cms/?page=trimmomatic: accessed on 24 October 2023) [32] normalization being carried out. Statistical analysis was conducted using the fold change method and the exact test using edgeR for each comparison pair. The significant findings were chosen based on the condition that the absolute value of |fc| ≥ 2 and the raw *p*-value from the exact test was equal to 2. To group similar samples and contigs, hierarchical clustering and principal component analysis (PCA) were conducted for substantial lists. The results are visually represented using a heatmap and dendrogram.

### 2.7. Validation of the Genes

In order to validate the findings from Illumina sequencing, a selection of 17 differentially expressed genes from each treatment group were chosen for RT-qPCR analysis. When selecting these genes, we carefully considered important processes such as the ribosome, autophagy, peroxisome, and spliceosome pathways. Primer5 software (Premier Biosoft International, Palo Alto, CA, USA) was utilized to design the primers, as shown in Table S2. An internal control was used in the RT-qPCR analysis, specifically the *β-actin* gene. In order to remove any genomic DNA, 500 ng of RNA from each sample was quantified and treated with RQ1 RNase-Free DNase (Promega, Madison, WI, USA). With the help of a reverse transcriptase reagent kit (Prime ScriptTM RT reagent Kit, Takara), the RNA that was treated underwent conversion into cDNA. The RT-qPCR was performed using SYBR Premix Ex Taq II from Takara (Takara, Tokyo, Japan). The reactions were carried out in a 25 μL total volume, with 2.5 μL of diluted cDNA, 2.5 μL of each primer, and 12.5 μL of SYBR Green PCR Master Mix. The cycling profile was as follows: 95 °C for 15 min for polymerase activation followed by 40 cycles at 95 °C for 15 s, 55 °C for 30 s, and 70 °C for 30 s. This was then followed by activating the polymerase for a certain period of time. The Roche Light Cycler 480 Real-Time PCR System was utilized to process each sample in triplicate. The 2^−ΔΔCT^ method was utilized to analyze all the data [33]. An analysis of variance was conducted to compare the effect of different temperatures on gene expression in *U. prolifera* tissue. This was followed by Tukey’s multiple comparison tests to further examine the results. The statistical software SPSS 20.0 for Windows (IBM SPSS Inc., Armonk, NY, USA) was utilized to analyze significant differences. A *p*-value below 0.05 was considered statistically significant. 

## 3. Results

### 3.1. Transcriptome Analysis

Ten cDNA libraries were constructed from *U. prolifera*, resulting in the acquisition of 37.48 gigabytes of sequencing data. Subsequently, the Trinity program was utilized to perform de novo assembly, resulting in a final dataset of 1,399,886 unigenes. These unigenes had an average length of 560.65 bp and an N50 value of 736 bp, indicating a high quality (Table S3). These unigenes were annotated using seven publicly available databases. Out of all the unigenes, a total of 1,275,834 were annotated, which corresponds to 76.48% of the entire unigene population. A total of 962,732 unigenes were annotated to the NR database, 305,489 to the NT database, 532,357 to the UniProt database, 409,468 to the KO_EUK database, 886,915 to the EggNOG database, 692,061 to the Pfam database, and 607,975 to the GO database. These numbers represent 75.46%, 23.94%, 41.73%, 32.09%, 69.52%, 54.24%, and 47.65% of the unigenes, respectively (Table S4).

### 3.2. Analysis of DEGs and Identification of Temperature-Responsive Genes

Initially, a substantial difference was observed in the principal component analysis (PCA) score plot between the treatment and control groups (Figure S1). This suggested that there were notable transcriptomic changes between the treatment and control groups. A total of 81,729 unigenes were identified as significantly differentially expressed genes (DEGs) compared to the gene expression levels of the controls. Following exposure to high temperature and light stress, a total of 49,796 genes were identified as differentially expressed, with 26,253 genes up-regulated and 23,543 genes down-regulated in the comparison between the H24 vs. C group. Similarly, in the comparison between the H48 vs. C group, a total of 47,066 differentially expressed genes were detected, with 25,250 genes up-regulated and 21,816 genes down-regulated (Figure S2, Table 1). Following exposure to a moderately high temperature and light stressor, a total of 44,468 differentially expressed genes (23,210 up-regulated and 21,258 down-regulated) were identified in the comparison between the MH24 vs. C group. Similarly, in the comparison between MH48 vs. C, a total of 44,169 differentially expressed genes (23,180 up-regulated and 20,989 down-regulated) were identified (Figure S2, Table 1).

The number of genes in the enriched pathways was 1207 for H24 vs. C and 1166 for H48 vs. C group when subjected to high temperature and high light intensity stress, compared to 1115 for MH24 vs. C and 1108 for MH48 vs. C under medium-high temperature and light intensity stress (Table 2). The density distribution of the expression level in each differentially expressed gene (DEG) library, as determined by log_2_ (CPM+2), revealed that the expression level was comparable across the H24 vs. C, H48 vs. C, MH24 vs. C, and MH48 vs. C libraries (Figure S3). The global expression profiles of differentially expressed genes (DEGs) in each library were determined using hierarchical clustering and heatmap analysis (Figure S4).

### 3.3. Functional Classification

**Figure 1:**
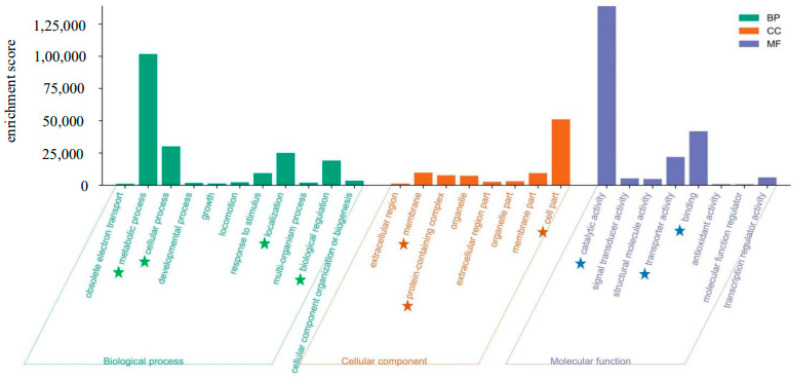
GO (Gene Ontology) categorization (biological process, cellular component, and molecular function) of the unigenes in the transcriptome of *Ulva prolifera*. Green star, key biological component; Orange star, key cellular component; Blue star, key molecular component.

To present a comprehensive summary of the various functional categories in the *U. prolifera* transcriptome database, the sequences were annotated with Gene Ontology (GO) terminology. GO analysis was performed to elucidate the molecular functions, cellular components, and biological processes associated with the adaptation of *U. prolifera* to high and medium-high temperature and light conditions. In total of 607,975 unigenes were annotated with one or more Gene Ontology (GO) concepts. Of them, 19,214 unigenes were annotated with molecular functions (30.84%), 17967 were assigned biological processes (28.84%), and 9287 were assigned cellular components (14.91%) (Table 2). The key cellular component types identified were cell part (GO:0044464), membrane (GO:0016020), and protein-containing complexes (GO:0032991). The key biological processes consisted of metabolic processes (GO:0008152), cellular processes (GO:0009987), localization (GO:0051179), and biological regulation (GO:0065007). The molecular functions that were most prominently represented include catalytic activity (GO:0003824), binding (GO:0005488), and transporter activity (GO:0005215) (Figure 1).

**Figure 2:**
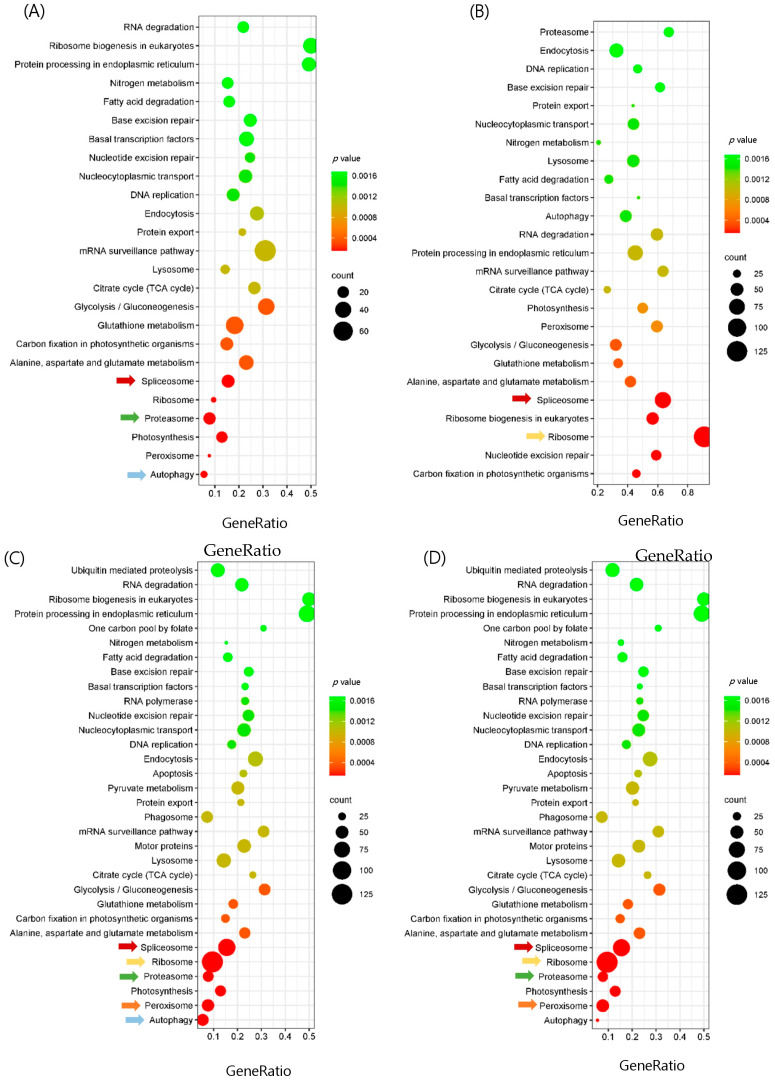
KEGG (Kyoto Encyclopedia of Genes and Genomes) assignment of unigenes in the transcriptome of *Ulva prolifera* between (**A**) H24 vs. C; (**B**) H48 vs. C; (**C**) MH24 vs. C; (**D**) MH48. Arrow: the most highly up-regulated pathway in different comparison groups. red arrow: Spliceosome pathway; yellow arrow: Ribosome pathway; green arrow: Proteasome pathway; Orange arrow: Peroxisome pathway; blue arrow: Autophagy pathway. Count indicates the number of differential genes annotated to the KEGG number. The color bar in *p*-value represents the significance of enrichment. The red dot size indicates highly enriched unigenes.

The genes were annotated and classified according to KEGG categories (Figure 2). An investigation using the KEGG pathway was conducted to determine the specific metabolic pathways that are active in *U. prolifera*. The 409,468 unigenes were categorized into 424 distinct pathways, encompassing metabolism, genetic information processing, environmental information processing, cellular processes, and organismal systems. The metabolism category had the highest number of unigenes, which were classified into nine main subgroups: carbohydrate metabolism, energy metabolism, lipid metabolism, amino acid metabolism, metabolism of other amino acids, glycan biosynthesis and metabolism, metabolism of cofactors and vitamins, biosynthesis of other secondary metabolites, and xenobiotics biodegradation and metabolism (Table S5). Among all the pathways, glycolysis/gluconeogenesis, pyruvate metabolism, citrate cycle (TCA cycle), pentose phosphate pathway, carbon fixation in photosynthetic organisms, and nitrogen metabolism were the most up-regulated pathways. Among the genetic information processing categories, ribosome, spliceosome, proteasome, and RNA degradation were the most up-regulated pathways. Among the cellular processes, lysosome, peroxisome, autophagy, and thermo-250 genesis were the most up-regulated pathways (Table S5).

Numerous genes (69.52%) were categorized into COG functional groups using EggNOG-mapper (version e5.proteomes) (Figure S5). The “function unknown” cluster was the biggest cluster found in the COG analysis. This was followed by the “cell wall/membrane/envelope biogenesis” cluster, the “amino acid transport and metabolism” cluster, the “energy production and conversion” cluster, and “signal transduction mechanisms”.

### 3.4. Enrichment and Pathway Analysis and Comparison of the Group

In this study, we focused on temperature and light-responsive pathways and genes that were enriched in both medium-high and high stress groups. In total, 434 pathways were enriched in the H24 vs. C and H48 vs. C groups, 432 in the MH24 vs. C group, and 431 in the MH48 vs. C group (Table 3). The medium-high and high stress groups showed similar expressions in most cases. Based on the KEGG database analysis, it was found that certain pathways, such as the ribosome, spliceosome, peroxisome, autophagy, and energy metabolism, had a higher number of up-regulated genes compared to other categories (Figure 2). As a result, our focus was directed toward exploring these pathways in more detail. From the transcriptome data, it was found that there was a significant enrichment of ribosome, spliceosome, and proteasome in both the medium and high stress groups. The KEGG spliceosome was significantly enriched in both high and medium-high temperature and light stress groups under both 24 h and 48 h (Figure 2).

In addition, ribosome-related genes were significantly up-regulated in the high stress group after 24 h and 48 h but only after 48 h under medium stress group (Figure 2). Similarly, proteasome-related genes were significantly up-regulated in the high stress group after 24 h and 48 h, whereas only after 24 h in the medium stress group (Figure 2). While autophagy-associated genes were significantly increased after 24 h in the high and medium-high stress groups but not after 48 h, peroxisome-related genes were only highly expressed in the high stress groups (Figure 2). Among these enriched pathways, genes involved in the spliceosome pathway were expressed in both the high and medium-high stress groups after 24 h and 48 h of stress, although the number of expressed genes varied between the populations.

We also found some pathways, for example, the glutathione pathway, alanine, aspartate and glutamate metabolism, and carbon fixation in photosynthetic organisms, which showed relatively low up-regulation but play a very significant role (Figure 2). These three pathways were up-regulated in all the comparison groups, but their expression was low.

### 3.5. Analysis of Major Pathway-Related Differentially Express Genes (DEGs) in Different Comparison Groups

Comparing DEGs in treatment groups with those in control groups, the effect of light intensity and temperature on the transcriptional regulation of *U. prolifera* was explored at different time points. Furthermore, the comparison of DEGs in treatment and control groups indicated the molecular mechanism of light and heat stress tolerance induced by *U. prolifera*.

Stress tolerance of *U. prolifera* is influenced by a number of genes. In all the comparison groups, the most similar number of DEGs was identified as ribosome-related DEGs and showed significant changes, and most of them were up-regulated compared to the control (Table 3). In the comparison between the H24 and C groups, 131 genes exhibited significant up-regulation or down-regulation. Similarly, in the H48 vs. C group, 130 genes showed significant changes in expression. For the MH24 vs. C group, 129 genes were significantly up-regulated or down-regulated, and in the MH48 vs. C group, 130 genes demonstrated notable alterations in expression, specifically encoding as ribosomal proteins when compared to the control (Table 3).

Likewise, the peroxisome and autophagy pathways exhibited a remarkably similar pattern of DEG expression across all comparison groups. In the H24 vs. C group, 47 genes displayed significant up-regulation or down-regulation. Similarly, in the H48 vs. C group, 48 genes showed significant changes in expression. For the MH24 vs. C group, 41 genes were significantly up-regulated or down-regulated, and in the MH48 vs. C group, 44 genes demonstrated notable alterations in expression, specifically encoding peroxisome-related genes compared to the control (Table 3).

Similarly, in the H24 vs. C group, 17 genes exhibited significant up-regulation or down-regulation. Likewise, in the H48 vs. C group, 16 genes showed significant changes in expression. For the MH24 vs. C group, 15 genes were significantly up-regulated or down-regulated, and in the MH48 vs. C group, 16 genes demonstrated notable alterations in expression, specifically encoding autophagy-related genes compared to the control (Table 3).

On the contrary, the spliceosome exhibited minimal variation in the number of DEGs. In the H24 vs. C group, 89 genes displayed significant up-regulation or down-regulation, while in the H48 vs. C group, 85 genes showed similar changes in expression. In both the MH24 vs. C and MH48 vs. C groups, 78 genes were significantly up-regulated or down-regulated compared to the control (Table 3).

### 3.6. Validation of RNA-seq Results with RT-qPCR

**Figure 3:**
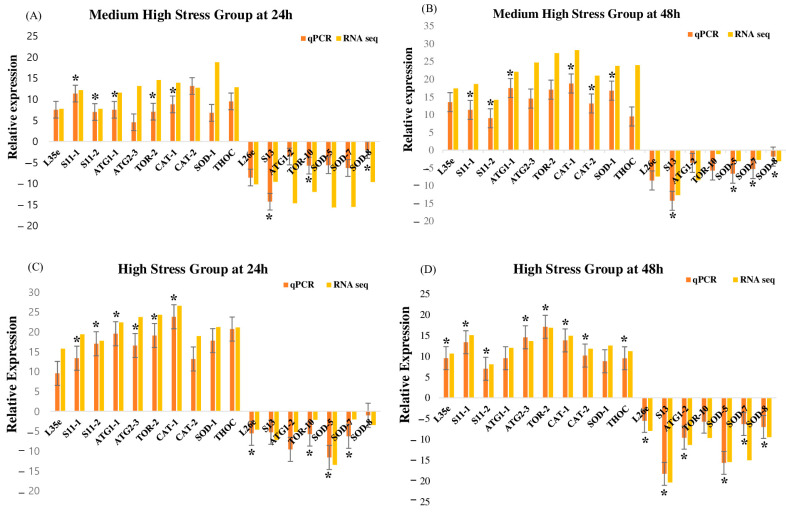
The relative mRNA expression pattern of different DEGs depending on different molecular functions under medium-high and high stress groups. (**A**) Medium-High Stress Group at 24 h; (**B**) Medium-High Stress Group at 48 h; (**C**) High Stress Group at 24 h; (**D**) High Stress Group at 48 h. The values are given in terms of relative mRNA expression. The transcript expression levels of the selected genes were normalized to that of the gene encoding β-actin. Data are presented as the means of replicates ± SE. Significant difference among samples is indicated by “*” (*p* < 0.05).

Seventeen genes were chosen depending on their functions in order to validate the patterns of DEGs found in the RNA-seq expression study which showed that the ribosome pathway was linked to five genes (*L35e*, *S11-1*, *S11-2*, *L26e*, and *S13*), the autophagy pathway was linked to five genes (*ATG1-1*, *ATG1-2*, *ATG2-3*, *TOR-2*, and *TOR-10*), the peroxisome pathway was linked to six genes (*CAT-1*, *CAT-2*, *SOD-1*, *SOD-5*, *SOD-7*, and *SOD-8*) and the spliceosome pathway was linked to one gene (*THOC*) (Table S6). The majority of the RT-qPCR results significantly (*p* < 0.05) showed a strong correlation with the RNA-seq results, according to our findings (Figure 3). *S11-1*, *S11-2*, *ATG1-1*, *TOR-2*, *S13*, *TOR-10*, *CAT-1*, and *SOD-8* were among the chosen genes that showed a significant up-regulation or down-regulation at 24 h under the medium-high stress group (Figure 3A), *S11-1*, *S11-2*, *ATG1-1*, *CAT-1*, *CAT-2*, *SOD-1*, *S13*, *SOD-5*, *SOD-7*, and *SOD-8* were significantly up-regulated or down-regulated under the medium-high stress group at 48 h (Figure 3B), *S11-1*, *S11-2*, *ATG1-1*, *ATG2-3*, *TOR-2*, *CAT-1*, *L26e*, *TOR-10*, *SOD-5*, and *SOD-7* were significantly up-regulated or down-regulated under the high stress group at 24 h (Figure 3C); *L35e*, *S11-1*, *S11-2*, *ATG2-3*, *S13*, *TOR-2*, *CAT-1*, *CAT-2*, *THOC*, *L26e*, *ATG1-2*, *SOD-5*, *SOD-7*, and *SOD-8* were significantly up-regulated or down-regulated under the high stress group at 48 h. According to Figure 3, the medium-high stress group had the lowest number of genes that were significantly elevated at 24 h, whereas the high stress group had the highest number of genes that were significantly up-regulated at 48 h, whereas *SOD-8*, *CAT-1*, *S11-1*, and *S11-2* were all considerably elevated across all groups. These genes’ expression levels altered dramatically in response to heat and light exposure, according to both RNA-seq data and RT-qPCR results. The great accuracy of the RNA-seq analysis was demonstrated by the RT-qPCR results that supported the RNA-seq results.

## 4. Discussion

In recent decades, the marine environment has changed due to different anthropogenic reasons [34,35]. Marine organisms often face stressful conditions caused by fluctuations in environmental factors like temperature, pH, pressure, shear force, and salinity. These conditions can lead to DNA damage and the production of reactive oxygen species (ROS) [36,37,38,39,40,41]. Furthermore, the presence of diverse habitats in the surrounding environment leads to variations in the capacity to withstand stress [38,39,42]. Hence, it can be inferred that fluctuations in the surrounding environmental conditions significantly influence the genetic diversity in *U. prolifera* and, consequently, lead to distinct adaptations in its biology [43,44,45].

### 4.1. Ribosome Metabolism

**Figure 4:**
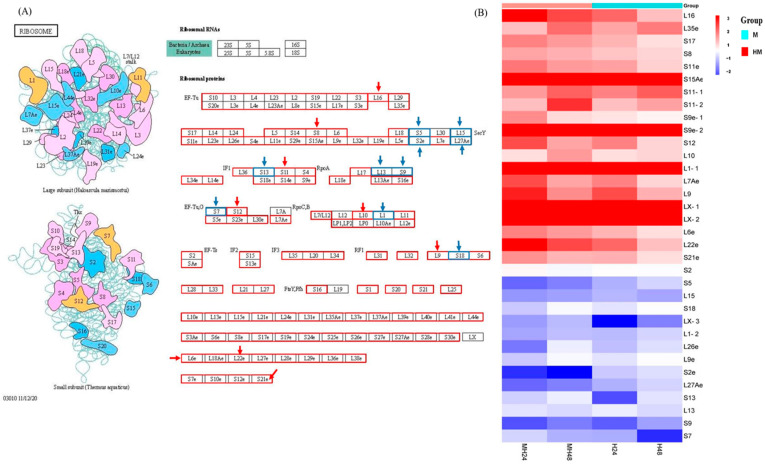
(**A**) Ribosome pathway. (**B**) Significantly up-regulated or down-regulated genes at the ribosome pathway. Here, red arrow means up regulated genes, blue arrow means down regulated genes.

The transcriptome data obtained from the present study indicated that the ribosome experiences the most severe damage under conditions of high and medium-high temperature and light stress, as depicted in Figure 4. Accelerated ribosomal protein synthesis may be required under high temperature/light stress in order to repair stress-induced damage and synthesize certain proteins that increase stress resistance [46,47]. Some of these genes and proteins are classified as Elongation Factors (EF) and play a significant role in responding to abiotic stress [48]. According to the studies conducted by Fu et al. (2012) and Chen and Cheng (2012) [48,49], EF-Tu genes are activated by abiotic stimuli and play a significant role in responding to stress. Research has shown that the excessive production of EF-Tu resulted in increased safeguarding of proteins from thermal aggregation, less harm to thylakoid membranes, and better photosynthetic performance after being exposed to abiotic stress [48,50]. EF-Tu forms a complex with GTP and aminoacyl tRNA, precisely placing it at the A site of the ribosome, breaking down GTP, and detaching EF-Tu-GDP from the ribosome [51]. EF-Tu has two main functions. Firstly, it transports the aminoacyl–tRNA complex to the A site of the ribosome during protein biosynthesis. Secondly, it functions as a chaperone to prevent environmental stress-induced protein aggregation and aids protein renaturation when conditions return to normal [48,52]. Ribosomal activity alterations are a component of this adaptation, guaranteeing modifications to sustain vital functions and endure in variable and frequently severe aquatic settings. The results indicated a strong correlation between ribosomal EF-Tu expression with *U. prolifera’s* abiotic stress tolerance and suggested that it is possible to enhance this tolerance by regulating the expression levels of EF-Tu.

### 4.2. Spliceosome Pathway

**Figure 5:**
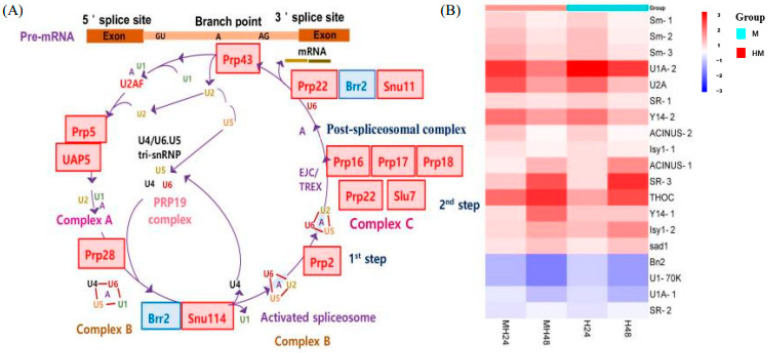
(**A**) Spliceosome pathway. (**B**) Significantly up-regulated or down-regulated genes at the spliceosome pathway.

Under conditions of combined temperature/light stress, one of the most harmful consequences was the induction of the spliceosome pathway in *U. prolifera*. RNA splicing is a crucial mechanism involved in gene expression in eukaryotes. The entire mechanism could be categorized into four distinct stages: spliceosome assembly, spliceosome activation, catalysis, and spliceosome disassembly. The mechanism in *Ulva* involved five snRNAs (small nuclear RNAs), including U1, U2, U4, U5, and U6, along with several other protein components (Figure 5). The process of spliceosome construction entailed sequential interactions between small nuclear ribonucleoproteins (snRNPs) and pre-messenger RNA (pre-mRNA). Spliceosome assembly began with UAP57 and Prp5 [53]. Prp5 also helped U2 bind to the spliceosome and become functional [54,55]. The activation of the spliceosome commences with the liberation of U1 and U2, succeeded by the attachment of the A complex. Snu114 and Brr2 were necessary to separate U4/U6 helices and release U4 from the spliceosome [56,57,58]. Snu114 is a GTPase that shares a high degree of similarity with translation elongation factor G [54]. It has been proposed to play a role in the activation of spliceosomes [59,60,61,62]. Prp2 and Prp16 participated in catalytic processes [57,58]. Prp2 liberates complex B after spliceosome activation [63,64], while Prp16 liberates complex C. Following the occurrence of the two catalytic events, the disassembly of the spliceosome necessitates the involvement of two EJC/TREX proteins. While Prp43 eventually causes the spliceosome that stores the intron to break apart, Prp22 first helps fully formed mRNA escape the spliceosome [65,66]. For modulating gene expression and preserving cellular homeostasis, the entire system is necessary. Algae are better able to handle stress because of these systems, which enable quick and adaptable gene expression responses to changes in the surrounding environment [67]. Spliceosome component alterations can produce distinct mRNA isoforms and activate transcription factors that may be more adapted to stressful environments, increasing the adaptability of the organism. These elements trigger a stress response mechanism that protects cellular integrity by causing drastic changes in gene expression and assisting cells in transitioning into a state akin to senescence, which allows them to withstand harsh environments until normal circumstances are restored [68]. These systems are strongly influenced by abiotic stress and involve a large number of stress-related genes. These genes are the primary contributors to stress signaling [50].

### 4.3. Peroxisome Metabolism

**Figure 6:**
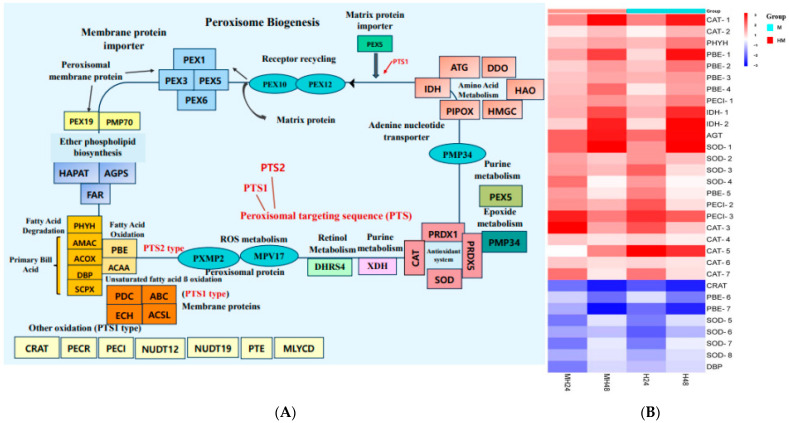
(**A**) Peroxisome pathway. (**B**) Significantly up-regulated or down-regulated genes at the peroxisome pathway.

The outcome of our study indicated that enhanced stress resistance could be a result of the proteins called peroxins (PEXs), which are involved in peroxisome biogenesis and are in charge of creating and maintaining peroxisomes. The overexpression of PEX genes ensures a sufficient supply of functional peroxisomes during stressful situations. PEX genes encode proteins necessary for the development and maintenance of peroxisomes [69]. The process of peroxisome formation begins with the initial peroxins PEX3, PEX16, and PEX19 and progresses through multiple stages. PEX19 is required for the identification, targeting, and insertion of membrane proteins into peroxisomes through docking with PEX3 (Figure 6). In *U. prolifera*, peroxisomal membrane proteins (PMPs) serve as chaperones for PMPs, specifically PEX3, and act as membrane anchors for PEX19 and PEX70. PEX70 is responsible for recruiting PEX3 to the endoplasmic reticulum (ER) prior to the formation of peroxisomes [70]. Peroxisomal targeting signals (PTS) identify matrix proteins in the cytosol and facilitate their transportation to the docking complex located at the peroxisomal membrane. The transportation of peroxisomal matrix proteins, which have C-terminal and N-terminal targeting signals known as PTS1 and PTS2, respectively, occurs through the soluble receptors PEX5 (for PTS1) in the cytosol [71,72]. Peroxisomes are vital organelles that have a crucial function in redox signaling and lipid balance [73]. ROS, which are detrimental byproducts of cellular metabolism that are amplified during stress, are neutralized by antioxidant enzymes found in peroxisomes. [74,75]. Furthermore, peroxisomal ROS have the ability to function as signaling molecules to trigger retrograde signaling, which activates stress response pathways and aids in the cell’s ability to adapt and endure harsh environments [75,76]. They are important for enhancing a number of vital metabolic processes, including the oxidation of fatty acids, the synthesis of ether lipids, the elimination of free radicals, and the effective transduction of stress signals (Figure 6). These modifications support overall stress tolerance by preserving cellular homeostasis and guarding against oxidative damage.

### 4.4. Autophagy Pathway

**Figure 7:**
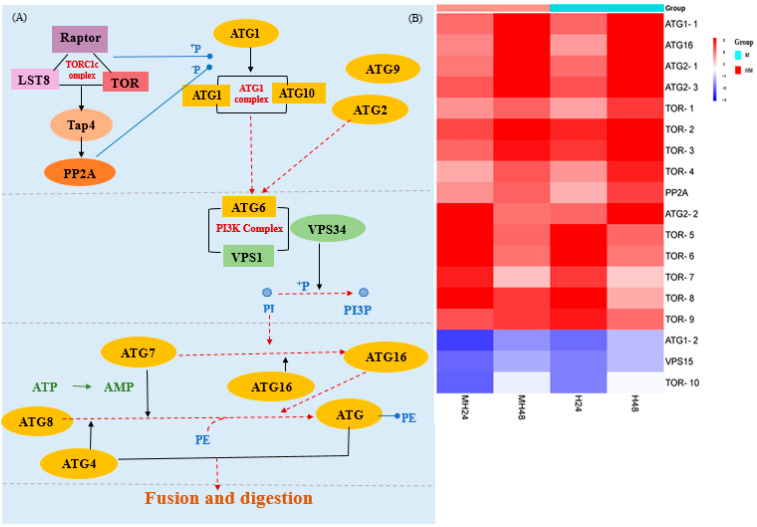
(**A**) Autophagy pathway. (**B**) Significantly up-regulated or down-regulated genes at the autophagy pathway.

The temperature in maritime habitats can significantly impact the functioning of immune defense mechanisms in macroalgae [77,78,79]. Significantly, we observed a notable increase in the expression of genes associated with autophagy following exposure to temperature/light stress in all the groups being compared (Figure 7). Elevated temperature/light stress can cause the buildup of bacterial infection in the tissues, leading to stress [80,81,82,83,84], diseases, and mortality [79]. Moreover, an indication of the immunological reaction of macroalgae against abiotic stresses is the increase in numerous antioxidant enzyme activities [85]. Studies have found stress triggered the production of reactive oxygen species (ROS) and other harmful molecules such as superoxide radicals, hydroxyl radicals, and hydrogen peroxide [86]. It also leads to alterations in various physiological, metabolic, and immune factors [84,85,87].

Autophagy can facilitate the recycling of damaged molecules, as well as organelles, including mitochondria, chloroplasts, and peroxisomes, hence enhancing stress tolerance [88,89,90]. In *U. prolifera*, autophagy initiation occurs at a specific perivacuolar location known as the induction site, which is located close to the vacuole. The activation of the *Atg1* protein complex, consisting of *Atg1*, *Atg13*, and *Atg101* [91,92], is controlled by TOR. During periods of stress, the deactivation of TOR leads to the removal of phosphate groups from *Atg13*, which enhances the creation of autophagosomes [93,94]. During the nucleation step (step 2), the Atg14-containing class III phosphatidylinositol (PtdIns) 3-kinase (PI3K) complex I, which included *Atg6* and *VPS15*, was brought to the PAS (Figure 7) [95]. In Stage 3, the phagophore initiated expansion and subsequently closed to the fully formed autophagosome. The key components involved in this step included *Atg7*, *Atg8*, *Atg4*, and *Atg16*. This mechanism was involved in the process of recruiting membranes to increase the phagophore. *Atg8* was activated through the assistance of *Atg7* and thereafter transported to *Atg4*. It then went through conjugation with the lipid phosphatidylethanolamine (PE), enabling its connection and fusion with the membrane. After reaching maturity, the autophagosome completely encloses the cargo and then merges with the vacuolar membrane to transport the cargo to the vacuole. The findings collectively indicated that alterations in water temperature played a pivotal role in governing immune-related factors, hence corroborating the outcomes of prior investigations [85,96,97].

### 4.5. Energy Metabolism

We speculated that increased energy production would follow changes in amino acid concentrations in order to keep the supply and demand of energy in balance. We speculated that despite the high energy demand, *U. prolifera* may sustain energy homeostasis by improving the metabolism of carbohydrates, amino acids, and lipids under 27 °C heat stress, suggesting that *U. prolifera* is capable of thermal stress adaptation. Obata and Fernie (2012) [98] found that under severe conditions, the enzymes responsible for glycolysis/gluconeogenesis, lipid metabolism, and photosynthesis were more abundant in macroalgae. Typically, when macroalgae are exposed to environmental stress, they respond by increasing the expression of genes related to energy production. This leads to a boost in carbohydrate metabolism pathways and the activation of alternative pathways like glycolysis and lipid metabolism. These changes provide the necessary energy for vital survival processes [51,99]. The current study revealed an increase in metabolic activity, specifically in glycolysis/gluconeogenesis, amino acid metabolism, lipid metabolism, photosynthesis, and carbon fixation in photosynthetic organisms. *U. prolifera* may possess the ability to tolerate fluctuations in temperature by regulating their amino acid levels and increasing energy production to maintain a balance between energy availability and demand.

### 4.6. Glutathione Pathway

Due to its antioxidant properties, glutathione is essential in helping seaweeds cope with high temperatures [100]. Elevated temperatures have the potential to escalate the generation of reactive oxygen species (ROS), hence inflicting substantial oxidative harm on cellular constituents such as proteins, DNA, and lipids. Several genes, including Tyr and PepA, were shown to be elevated during stressful conditions in our study. These genes directly scavenge reactive oxygen species (ROS) to prevent damage to cellular components. Because oxidative stress is increased in seaweeds exposed to high temperatures and light levels, this antioxidant effect is especially significant [101].

### 4.7. Carbon Fixation in Photosynthetic Organisms

Carbon fixation in seaweeds is a dynamic process that plays a critical role in their response to high-temperature stress. Seaweeds can withstand harsh heat conditions and maintain growth and productivity by optimizing carbon fixation and related metabolic pathways. Because of climate change, their capacity to survive in more unpredictable marine habitats is crucial [34]. According to our research, several genes related to carbon fixation in photosynthetic organisms heat stress were up-regulated, which means an increase in the expression of genes linked to carbon fixation, indicating a coordinated reaction to preserve metabolic balance and guard against thermal damage in macroalgae [34].

### 4.8. Alanine, Aspartate, and Glutamate Metabolism

Our recent research demonstrated that heat stress causes many seaweeds to up-regulate their alanine, aspartate, and glutamate metabolic pathways. The organisms’ ability to sustain nitrogen metabolism, osmotic stability, and energy balance is essential for life in high-temperature environments which is aided by these metabolic modifications. Our research showed that heat stress alters the metabolism of amino acids, resulting in elevated alanine and glutamate levels [34]. This suggests that these amino acids have a role in energy production, stress reduction, and strengthening resistance to the consequences of global warming.

### 4.9. Differentially Expressed Genes

Among all the significantly up-regulated or down-regulated DEGs, four genes (*SOD-8*, *CAT-1*, *S11-1*, and *S11-2*) were identified in all the comparison groups, which highlights the potential importance of these four genes in the stress resistance mechanisms of *U. prolifera* [101]. This suggests that these four genes may play a crucial role in the stress resistance mechanisms of *U. prolifera*. These genes could potentially play a role in essential biological processes or pathways associated with abiotic stress. By comprehending their roles, valuable insights can be gained into the underlying mechanisms of *U. prolifera’s* stress tolerance. Additionally, these roles can serve as a dependable biomarker for studies related to stress [102].

Studying the adaptation of *U. prolifera* to environmental change has significant implications for the advancement of more productive and sustainable macroalgae culture [2,14]. Transcriptomic studies have proven to be valuable in identifying instances of positive selection across various species. This knowledge can then be leveraged to employ gene editing techniques in order to cultivate specific desired traits within the species of focus [103]. Gaining this valuable information will enhance our understanding of seaweed culture and conservation management [104]. Transcriptomic studies are valuable in understanding stress tolerance levels. The findings can be utilized to create a useful resource for enhancing the well-being and stress tolerance of macroalgae species [105]. In addition, transcriptome sequencing enhances genomic resources to enhance resistance to environmental stressors and promote the well-being of aquaculture species, which is crucial for the sustainable practice of macroalgae cultivation [9,105].

## 5. Conclusions

For this study, we analyzed the transcriptome of green macroalgae (*U. prolifera*) when exposed to different stress conditions. Our focus was on identifying stress-related genes and pathways. The analysis results demonstrated the variety of stress-responsive pathways and gene expression changes in response to the changing marine environment. These results provided important insights into the susceptibility of *U. prolifera* to different abiotic stress conditions and have the potential to estimate future genetic biodiversity in climate change scenarios. Future studies are necessary, focusing on specific genes and pathways to find out how changes in specific genes and pathways optimize the stressful conditions for survival and growth.

## Figures and Tables

**Table 1:** Gene expression differences in different comparison groups.

Group	Average Logcpm	Mean Fold Change Differentially Expressed Genes	Differentially Expressed Genes DEGs *p*-Value (Average)	DEG	Up-Regulated DEGs	Down-Regulated DEGs
H24 vs. C	1.177248	−6.896698	0.017459	49,796	26,253	23,543
H48 vs. C	0.983384	−14.470383	0.22426397	47,066	25,250	21,816
MH24 vs. C	1.276447	−20.981178	0.004089	44,468	23,210	21,258
MH48 vs. C	1.287536	−16.937280	0.001698	44,169	23,180	20,989

**Table 2:** Number and percentage of gene annotation.

GO Term	% Unigenes	Number of Unigenes
Molecular functions	30.84%	19,214
Biological processes	28.84%	17,967
Cellular components	14.91%	9287
Total unigenes		46,468

**Table 3:** Summary of transcriptomic data of the pathways and genes in different treatment groups.

	H24 vs. C	H48 vs. C	MH24 vs. C	MH48 vs. C
Total enriched pathway	434	434	432	431
Total enriched genes	1207	1166	1115	1108
Significantly up-regulated or down-regulated genes in ribosome pathway	131	130	129	130
Significantly up-regulated or down-regulated Genes in the peroxisome pathway	47	48	41	44
Significantly up-regulated or down-regulated genes in the autophagy pathway	17	16	15	16
Significantly up-regulated or down-regulated genes in spliceosome pathway	89	85	78	78

## Data Availability

Data will be available on the National Center for Biotechnology Information (NCBI) Short Read Archive (SRA) database with the assigned accession numbers of PRJNA1061775, PRJNA1061781, PRJNA1062022, PRJNA1092455, and PRJNA1062028.

## Supplementary Materials

The following supporting information can be downloaded at https://www.mdpi.com/article/10.3390/biology13090725/s1, Figure S1. Principal component analysis (PCA) plot of differentially expressed genes, H24, high temperature/light stress after 24 h; MH24, medium-high temperature/light stress after 24 h; H48, high temperature/light stress after 48 h; MH48, high temperature/light stress after 48 h; Figure S2. Differential Gene Expressions (DEGs) expression levels of the treatments compared with the control, H24, high temperature/light stress after 24 h; MH24, medium-high temperature/light stress after 24 h; H48, high temperature/light stress after 48 h; MH48, high temperature/light stress after 48 h; Figure S3. Volcano plot of differentially expressed genes. (A) H24 vs. C (B) H48 vs. C (C) MH24 vs. C (D) MH48 vs. C. H24, high temperature/light stress after 24 h; MH24, medium-high temperature/light stress after 24 h; H48, high temperature/light stress after 48 h; MH48, high temperature/light stress after 48 h; Figure S4. Hierarchical clustering of DEGs union among different comparisons DGEs libraries, H24, high temperature/light stress after 24 h; MH24, medium-high temperature/light stress after 24 h; H48, high temperature/light stress after 48 h; MH48, high temperature/light stress after 48 h; Figure S5. COG functional classification of *Ulva prolifera* genome: [J] translation, ribosomal structure, and biogenesis; [A] RNA processing and modification; [K] transcription; [L] replication, recombination, and repair; [B] chromatin structure and dynamics; [D] cell cycle control, cell division, chromosome partitioning; [Y] nuclear structure; [V] defense mechanisms; [T] signal transduction mechanisms; [M] cell wall/membrane/envelope biogenesis; [N] cell motility; [Z] cytoskeleton; [W] extracellular structures; [U] intracellular trafficking, secretion, and vesicular transport; [O] post-translational modification, protein turnover, and chaperones; [C] energy production and conversion; [G] carbohydrate transport and metabolism; [E] amino acid transport and metabolism; [F] nucleotide transport and metabolism; [H] coenzyme transport and metabolism; [I] lipid transport and metabolism; [P] inorganic ion transport and metabolism; [Q] secondary metabolites biosynthesis, transport, and catabolism; [R] general function prediction only; [S] function unknown; Table S1: Information and quality of RNA-seq; Table S2: Combination of primers used in RT-qPCR assays; Table S3: Summary statistics of transcriptome assembly; Table S4: Annotation result of various databases; Table S5: KEGG Pathway Mapping; Table S6: Validation of RNA-Seq results using RT–qPCR. The transcript expression levels of the selected genes were normalized to that of the β-actin gene.
