# Supplementary material for: Transcriptomic Analysis Provides New Insights into the Tolerance Mechanisms of Green Macroalgae Ulva prolifera to High Temperature and Light Stress
Source: Biology (Basel). 2024 Sep 16;13(9):725. doi: 10.3390/biology13090725 (PMC11428574; doi:10.3390/biology13090725)
Supplement: Supplementary file 1 [file biology-13-00725-s001.zip › Table S1.pdf]

Table S1 Information and quality of RNA-seq

Sample	Total Raw reads	Total Clean reads	Total Read Bases	Total Clean bases	Raw Q20(%)	Clean Q20(%)	Raw Q30(%)	Clean Q30 (%)	Raw GC (%)	Clean GC (%)
C_1	70,991,584	6 70,051,132	7,170,149,984	7,051,250,02	98.45	98.84	95.23	95.79	44.32	44.32
C_2	75,697,826	9 74,478,478	7,645,480,426	7,492,577,51	98.27	98.74	94.89	95.57	44.8	44.79
H24_1	72,861,134	7 72,053,644	7,358,974,534	7,251,124,84	98.55	98.88	95.39	95.87	42.94	42.94
H24_2	77,564,480	2 76,675,386	7,834,012,480	7,718,578,50	98.54	98.88	95.4	95.9	41.64	41.64
H48_1	74,134,242	1 73,177,794	7,487,558,442	7,363,726,99	98.4	98.79	95.07	95.63	43.05	43.04
H48_2	69,809,048	7 68,695,600	7,050,713,848	6,911,708,20	98.29	98.77	94.92	95.59	42.6	42.59
MH24_1	72,141,646	9 71,078,998	7,286,306,246	7,153,126,04	98.34	98.78	95.0	95.62	40.15	40.14
MH24_2	73,196,946	3 72,121,328	7,392,891,546	7,257,529,22	98.4	98.84	95.21	95.83	42.0	41.99
MH48_1	74,014,938	5 73,123,972	7,475,508,738	7,360,518,69	98.51	98.87	95.32	95.83	41.36	41.36
MH48_2	81,111,926	4 79,984,706	8,192,304,526	8,051,275,79	98.47	98.89	95.34	95.93	42.14	42.12
