# Supplementary material for: Transcriptomic Analysis Provides New Insights into the Tolerance Mechanisms of Green Macroalgae Ulva prolifera to High Temperature and Light Stress
Source: Biology (Basel). 2024 Sep 16;13(9):725. doi: 10.3390/biology13090725 (PMC11428574; doi:10.3390/biology13090725)
Supplement: Supplementary file 1 [file biology-13-00725-s001.zip › Table S2.pdf]

Table S2 Combination of primers used in RT-qPCR assays

Gene			Sequence
L35e	Ribosomal protein L35e	F	GCTCTCGCAACGATGAAGAA
		R	TGAACTCAGGTCGAATGAATGG
S11-1	Ribosomal protein S11 isoform 1	F	GCAGCCTTCTAAGCGAGTAA
		R	TGAACCCGCAAAGGAGAAA
S11-2	Ribosomal protein S11 isoform 2	F	AAGGCGAGGGTTTCAAAGAG
		R	CCTAGTTGGCTTGTCGCTATAC
L26e	Ribosomal protein L26e	F	CTGGAGTGATGAGCTGAAAGAA
		R	CACATAAGAAGGCGACCAAGA
S13	Ribosomal protein S13	F	GCTTTCTCGCTTTCTCCTTTG
		R	GCCTCCCTTGCTTTGAGATA
ATG1-1	Autophagy-related protein	F	GGCGATGAAGGACGTGATAA
		R	CACTATCGGTCTCTCAGGAGTA
ATG1-2	Autophagy-related protein	F	CGGCCGCATAACTTCGTATAG
		R	CTTCTCCTCCGGGTACTTAGAT
ATG2-3	Autophagy-related protein	F	GGACGTGATAAGCTGCGATAAG
		R	GGTCCCGCCAAATTCATACA
TOR-2	Threonine-protein kinase mTOR	F	GTCGTCGTAGCTGCAATCA
		R	TCTCGCTTTCTCGCTTTCTC
TOR-10	Threonine-protein kinase mTOR	F	GGCTTGGATCAGCAACCTAA
		R	CCTAGTTGGCTTGTCGCTATAC
CAT-1	Catalase	F	TCCTAGGGAATCCATCGAGTAG
		R	GCCTCCCTTGCTTTGAGATATAA
CAT-2	Catalase	F	TTAGCAGCGTTCCGTATGAC
		R	ACAACCTAATAAACCACGACCA
SOD-1	Superoxide dismutase	F	AGAATCTCTGTCCTGCAAAGG
		R	TGCAACATGAGCCACAGTAT
SOD-5	Superoxide dismutase	F	GGCAATCTTCTTGACGCAATC
		R	CCAGACATCAATGCCGAGAA
SOD-7	Superoxide dismutase	F	TCCTTGCTCAGGCAATCTTC
		R	CGTGCTCCCAGACATCAAT
SOD-8	Superoxide dismutase	F	GGATGCACATCCTTGCTCA
		R	GGATCCAATCCTGGTGCAA
THOC	THO complex	F	GTTCCGTATGACTCCTCAACC
		R	TCATAAACAGCACGTCCATAGT
