# Supplementary material for: Transcriptomic Analysis Provides New Insights into the Tolerance Mechanisms of Green Macroalgae Ulva prolifera to High Temperature and Light Stress
Source: Biology (Basel). 2024 Sep 16;13(9):725. doi: 10.3390/biology13090725 (PMC11428574; doi:10.3390/biology13090725)
Supplement: Supplementary file 1 [file biology-13-00725-s001.zip › Table S3.pdf]

Table S3 Summary statistics of transcriptome assembly

Assembly	merge	
	ALL transcript contigs	ONLY LONGEST ISOFORM per 'GENE'
Total trinity 'genes'	1,399,886	1,399,886
Total trinity transcripts	1,555,749	1,399,886
Percent GC	48.63	48.32
N90	252	245
N80	325	306
N70	434	393
N60	603	525
N50	865	736
N40	1,255	1,070
N30	1,815	1,585
N20	2,706	2,429
N10	4,513	4,186
Maximum contig length	58,257	58,257
Minimum contig length	201	201
Median contig length	334.0	323.0
Average contig length	604.35	560.65
Total assembled bases	940,210,770	784,843,675
