# Supplementary material for: Transcriptomic Analysis Provides New Insights into the Tolerance Mechanisms of Green Macroalgae Ulva prolifera to High Temperature and Light Stress
Source: Biology (Basel). 2024 Sep 16;13(9):725. doi: 10.3390/biology13090725 (PMC11428574; doi:10.3390/biology13090725)
Supplement: Supplementary file 1 [file biology-13-00725-s001.zip › Table S4.pdf]

Table S4 Annotation result of various databases

Assembly	Merge
Total Unigene	1,275,834
GO	607,975 (47.65%)
UniProt	532,357 (41.73%)
NR	962,732 (75.46%)
Pfam	692,061 (54.24%)
EggNOG	886,915 (69.52%)
NT	305,489 (23.94%)
KO_EUK	409,468 (32.09%)
Overall	975,794 (76.48%)
