# Supplementary material for: Transcriptomic Analysis Provides New Insights into the Tolerance Mechanisms of Green Macroalgae Ulva prolifera to High Temperature and Light Stress
Source: Biology (Basel). 2024 Sep 16;13(9):725. doi: 10.3390/biology13090725 (PMC11428574; doi:10.3390/biology13090725)
Supplement: Supplementary file 1 [file biology-13-00725-s001.zip › Table S5.pdf]

Table S5 KEGG Pathway Mapping

KEGG Pathway	Sub Pathway	
Metabolism	Carbohydrate Metabolism	Glycolysis / Gluconeogenesis
		Pyruvate metabolism
		Citrate cycle (TCA cycle)
		Pentose phosphate pathway
	Energy metabolism	Oxidative phosphorylation
		Photosynthesis
		Carbon fixation in photosynthetic organisms
		Nitrogen metabolism
	Lipid metabolism	Glycerolipid metabolism
		Glycerophospholipid metabolism
		Sphingolipid metabolism
	Amino acid metabolism	Alanine, aspartate and glutamate metabolism
		Glycine, serine and threonine metabolism
		Valine, leucine and isoleucine degradation
		Valine, leucine and isoleucine biosynthesis
		Arginine biosynthesis
		Arginine and proline metabolism
		Histidine metabolism
		Phenylalanine, tyrosine and tryptophan biosynthesis
	Metabolism of other amino acids	beta-Alanine metabolism
		Selenocompound metabolism
		Glutathione metabolism
	Glycan biosynthesis and metabolism	N-Glycan biosynthesis
		Various types of N-glycan biosynthesis
		Other types of O-glycan biosynthesis
		Glycosylphosphatidylinositol (GPI)-anchor biosynthesis
		Other glycan degradation
	Metabolism of cofactors and vitamins	Riboflavin metabolism
		Pantothenate and CoA biosynthesis
		One carbon pool by folate
	Biosynthesis of other secondary metabolites	Streptomycin biosynthesis
	Xenobiotics biodegradation and metabolism	Styrene degradation
		Drug metabolism - other enzymes
		Caprolactam degradation
Genetic Information Processing	Transcription and Translation	RNA polymerase
		Basal transcription factors
		Spliceosome
		Ribosome
		Nucleocytoplasmic transport

		mRNA surveillance pathway
		Ribosome biogenesis in eukaryotes
	Folding, sorting and degradation	Protein export
		Protein processing in endoplasmic reticulum
		Ubiquitin mediated proteolysis
		Proteasome
		RNA degradation
	Replication and repair	DNA replication
		Base excision repair
		Nucleotide excision repair
		Mismatch repair
		Non-homologous end-joining
Environmental Information Processing	Signal transduction	MAPK signaling pathway - plant
		Hedgehog signaling pathway - fly
		HIF-1 signaling pathway
		FoxO signaling pathway
		Phosphatidylinositol signaling system
		Sphingolipid signaling pathway
		AMPK signaling pathway
		mTOR signaling pathway
	Signaling molecules and interaction	ECM-receptor interaction
Cellular Processes	Transport and catabolism	Endocytosis
		Phagosome
		Lysosome
		Peroxisome
		Autophagy - other
	Cell growth and death	Cell cycle
		Oocyte meiosis
	Cell motility	Motor proteins
		Regulation of actin cytoskeleton
	Immune system	Antigen processing and presentation
		Fc gamma R-mediated phagocytosis
	Aging	Longevity regulating pathway
	Environmental adaptation	Circadian rhythm - plant
		Thermogenesis
