# Supplementary material for: Transcriptomic Analysis Provides New Insights into the Tolerance Mechanisms of Green Macroalgae Ulva prolifera to High Temperature and Light Stress
Source: Biology (Basel). 2024 Sep 16;13(9):725. doi: 10.3390/biology13090725 (PMC11428574; doi:10.3390/biology13090725)
Supplement: Supplementary file 1 [file biology-13-00725-s001.zip › Table S6.pdf]

Table S6 Validation of RNA-Seq results using RT–qPCR. The transcript expression levels of the selected genes were normalized to that of the β-actin gene

Group	MH24		MH48		H24		H48	
Gene	qPCR	RNA seq	qPCR	RNA seq	qPCR	RNA seq	qPCR	RNA seq
L35e	7.533483	7.739186	13.53348	17.41807	9.533483	15.75086	9.533483	10.61029
S11-1	11.35866	12.16542	11.35866	18.63431	13.35866	19.37928	13.35866	15.08288
S11-2	6.987485	7.74838	8.987485	14.19106	16.98749	17.73506	6.987485	8.048188
ATG1-1	7.511475	11.57043	17.51148	22.08575	19.51148	22.33658	9.511475	12.01042
ATG2-3	4.548325	13.16034	14.54833	24.73665	16.54833	23.72984	14.54833	13.62427
TOR-2	7.064529	14.56402	17.06453	27.33606	19.06453	24.26378	17.06453	16.85427
CAT-1	8.793853	13.92665	18.79385	28.21543	23.79385	26.55444	13.79385	14.90553
CAT-2	13.15716	12.73861	13.15716	20.98513	13.15716	18.91057	10.15716	11.79279
SOD-1	6.782211	18.79174	16.78221	23.79529	17.78221	21.18751	8.782211	12.55133
THOC	9.504401	12.84751	9.504401	23.96204	20.69131	21.0969	9.504401	11.2033
L26e	-8.54652	-10.1336	-8.54652	-7.39238	-5.54652	-4.62323	-5.54652	-7.93145
S13	-14.2777	-9.55469	-14.2777	-12.6517	-5.27767	-7.20169	-18.2777	-20.339
ATG1-2	-3.58111	-14.6685	-3.58111	-8.46962	-9.58111	-5.20809	-9.58111	-11.3065
TOR-10	-5.7345	-11.9628	-5.7345	-1.11456	-5.7345	-2.13166	-5.7345	-9.64002
SOD-5	-5.62961	-15.6311	-6.62961	-3.09134	-11.6296	-13.453	-15.6296	-15.4394
SOD-7	-6.29978	-15.5012	-5.29978	-2.70744	-6.29978	-2.0189	-6.29978	-14.9904
SOD-8	-2.0115	-9.6311	-1.8115	-3.09134	-1.0115	-3.45302	-7.0115	-9.4394
