# Supplementary figures and images for: Transcriptomic Analysis Provides New Insights into the Tolerance Mechanisms of Green Macroalgae Ulva prolifera to High Temperature and Light Stress
Source: Biology (Basel). 2024 Sep 16;13(9):725. doi: 10.3390/biology13090725 (PMC11428574; doi:10.3390/biology13090725)

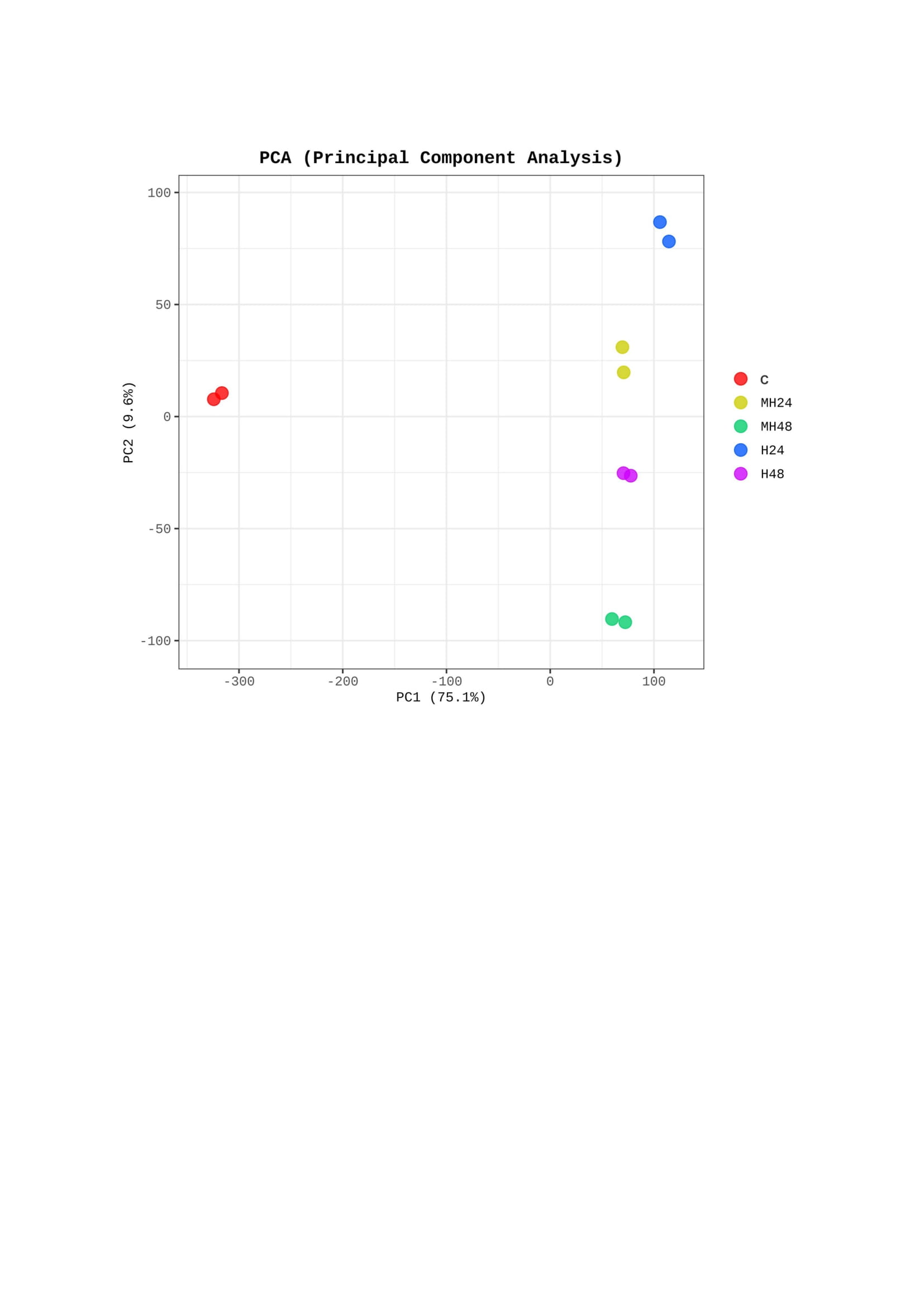

Supplement: Supplementary file 1 [file biology-13-00725-s001.zip › Figure S1.jpg]

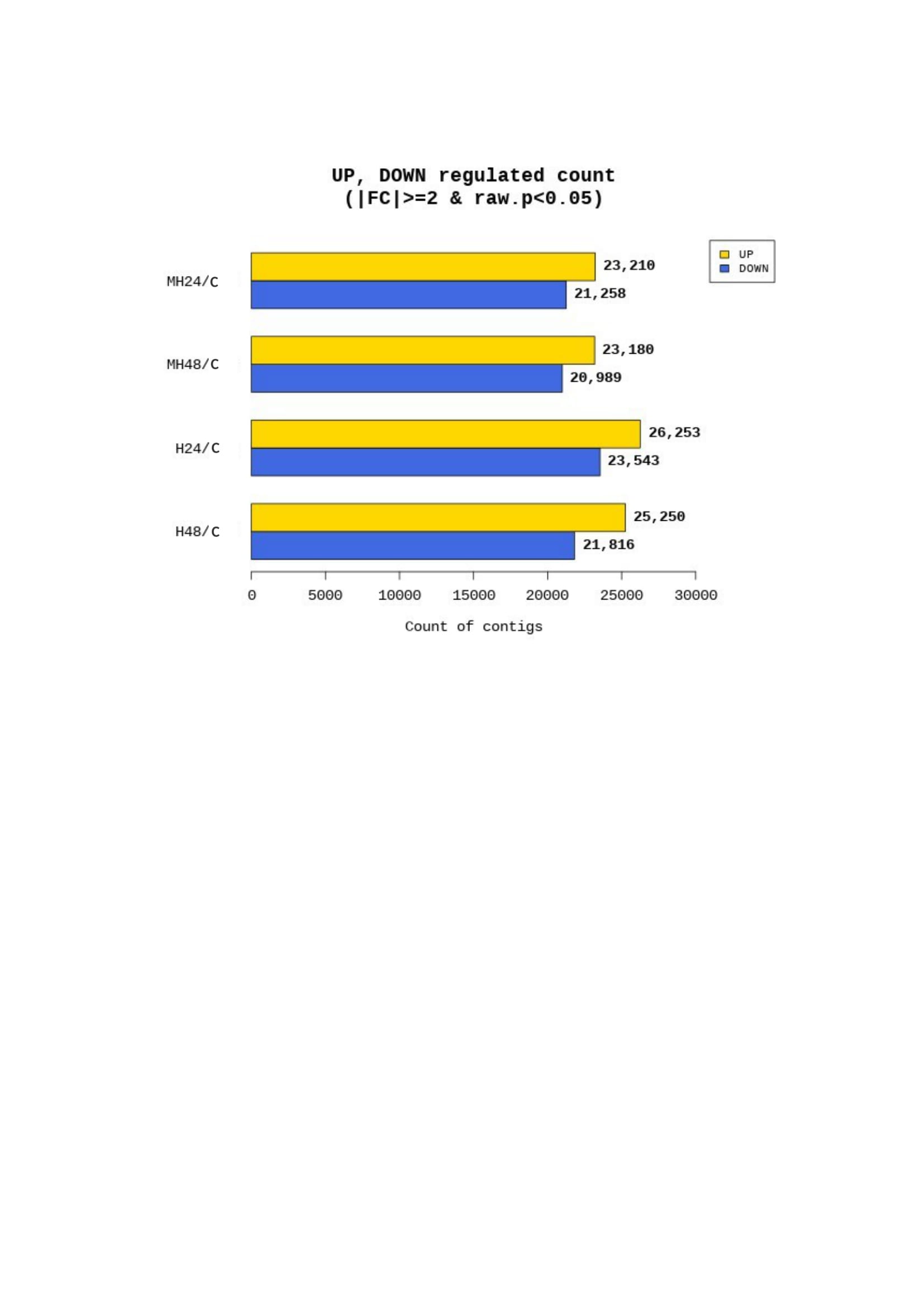

Supplement: Supplementary file 1 [file biology-13-00725-s001.zip › Figure S2.jpg]

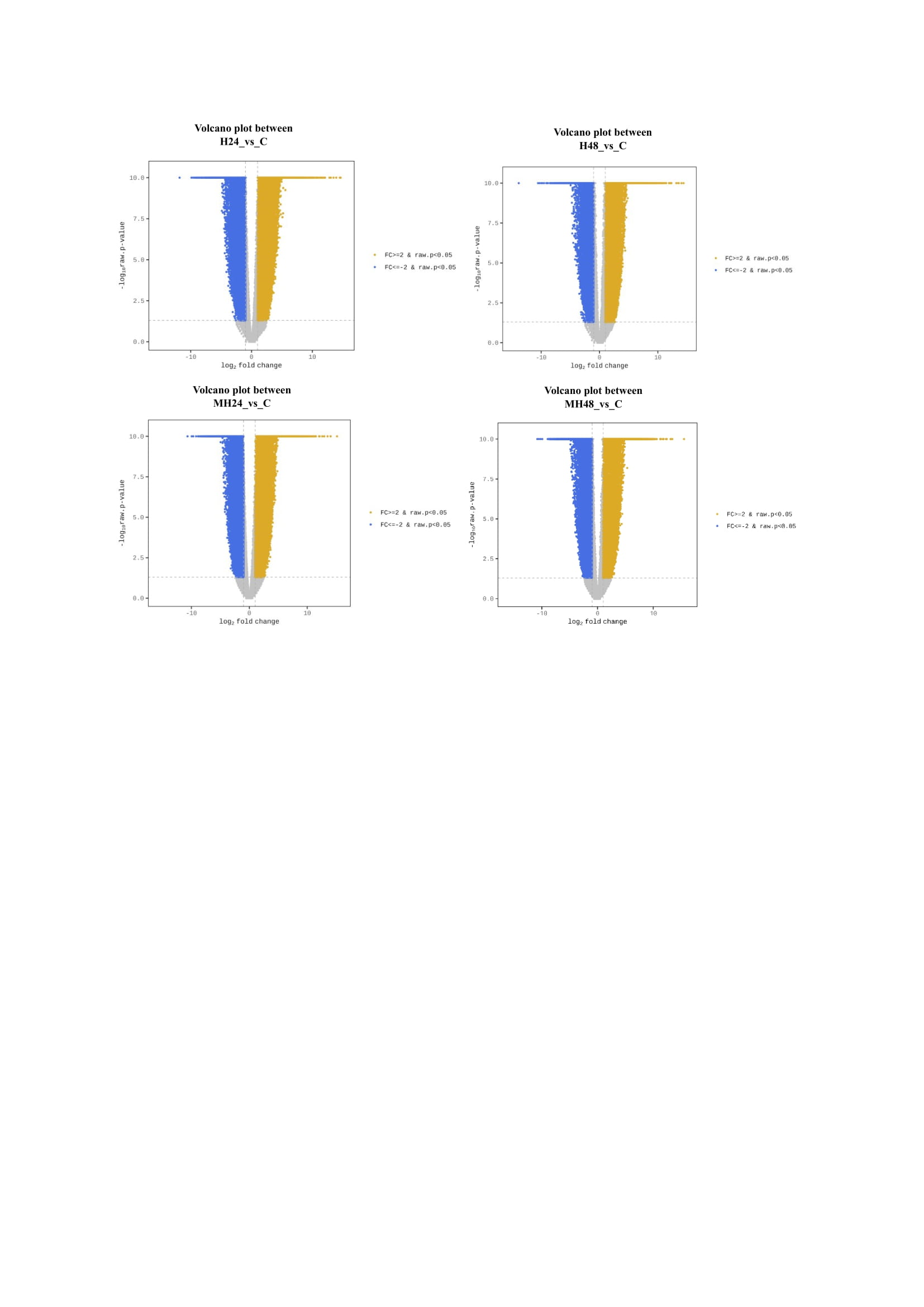

Supplement: Supplementary file 1 [file biology-13-00725-s001.zip › Figure S3.jpg]

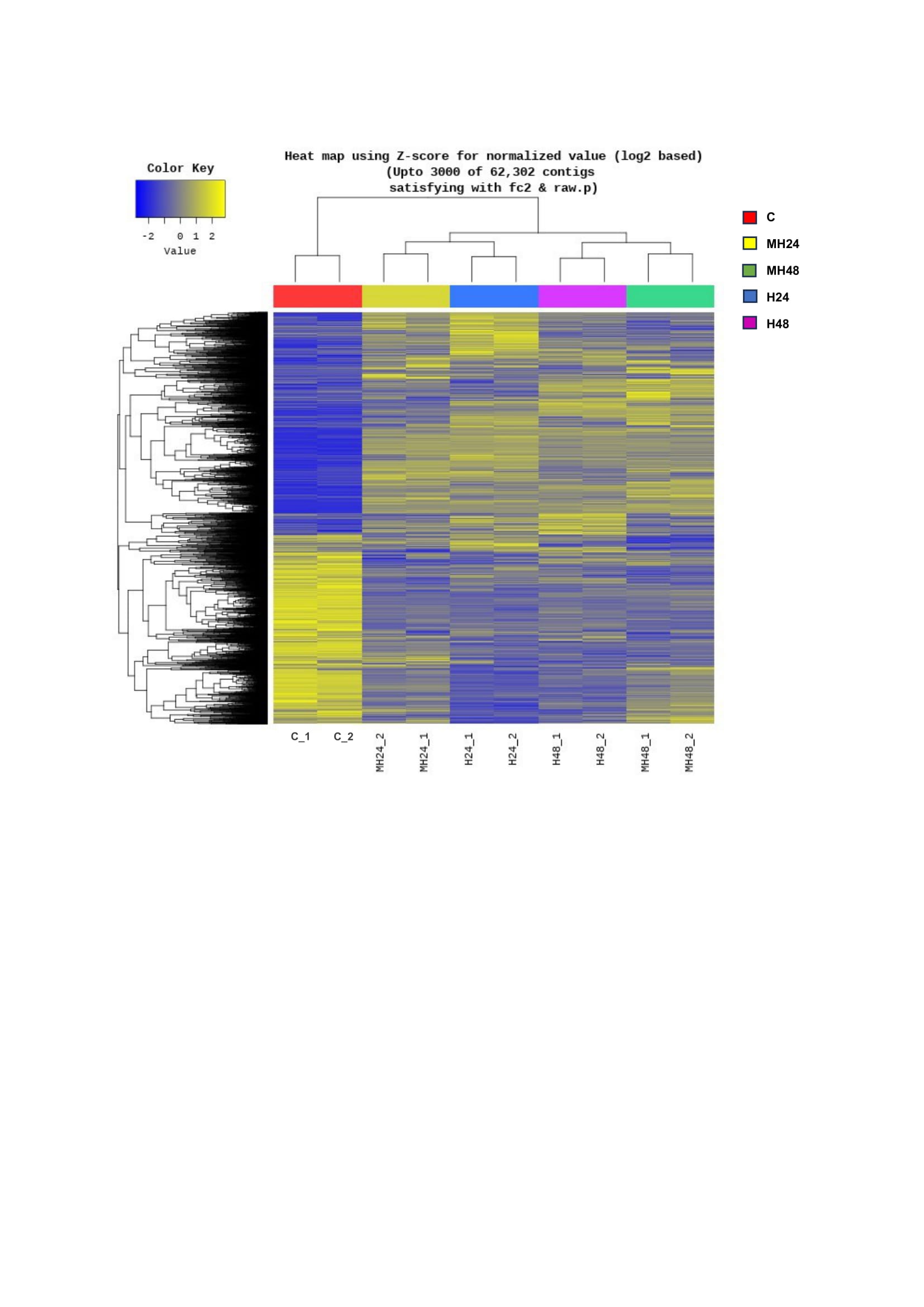

Supplement: Supplementary file 1 [file biology-13-00725-s001.zip › Figure S4.jpg]

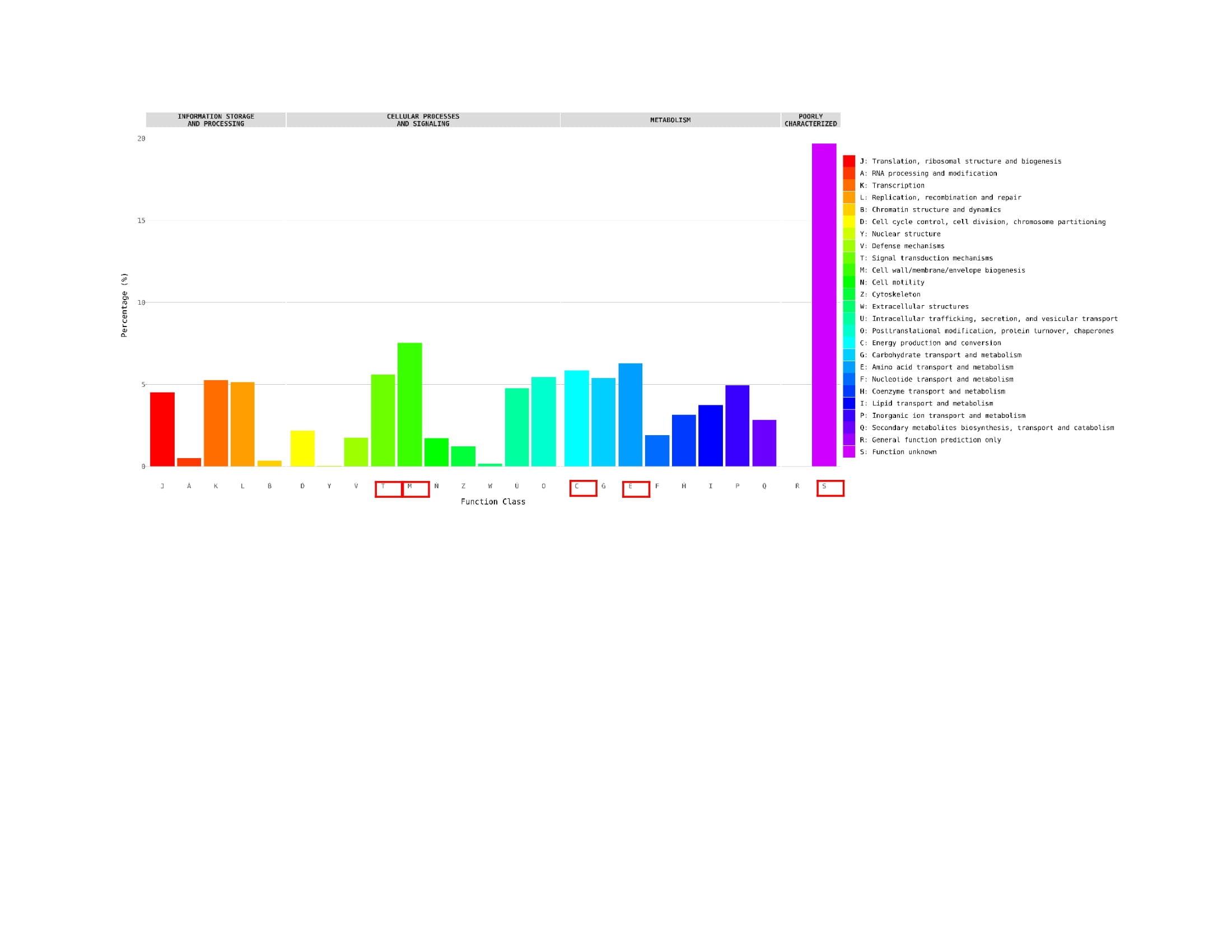

Supplement: Supplementary file 1 [file biology-13-00725-s001.zip › Figure S5.jpg]
